# Benzyl isothiocyanate improves the prognosis of *Aspergillus fumigatus* keratitis by reducing fungal load and inhibiting Mincle signal pathway

**DOI:** 10.3389/fmicb.2023.1119568

**Published:** 2023-02-16

**Authors:** Wendan Yi, Lingwen Gu, Yuwei Wang, Jing Lin, Lina Zhang, Qian Wang, Weilin Diao, Yinghe Qi, Menghui Chi, Min Yin, Cui Li, Guiqiu Zhao

**Affiliations:** Department of Ophthalmology, The Affiliated Hospital of Qingdao University, Qingdao, China

**Keywords:** benzyl isothiocyanate, *Aspergillus fumigatus* keratitis, antifungal, anti-inflammatory, Mincle

## Abstract

*Aspergillus fumigatus* keratitis is a potential blinding disease associated with *A. fumigatus* invasion and excessive inflammatory response. Benzyl isothiocyanate (BITC) is a secondary metabolite with broad antibacterial and anti-inflammatory activity extracted from cruciferous species. However, the role of BITC in *A. fumigatus* keratitis has not been discovered yet. This study aims to explore the antifungal and anti-inflammatory effects and mechanisms of BITC in *A. fumigatus* keratitis. Our results provided evidences that BITC exerted antifungal effects against *A. fumigatus* by damaging cell membranes, mitochondria, adhesion, and biofilms in a concentration-dependent manner. *In vivo*, fungal load and inflammatory response including inflammatory cell infiltration and pro-inflammatory cytokine expression were reduced in BITC-treated *A. fumigatus* keratitis. Additionally, BITC significantly decreased Mincle, IL-1β, TNF-α, and IL-6 expression in RAW264.7 cells that stimulated by *A. fumigatus* or Mincle ligand trehalose-6,6-dibehenate. In summary, BITC possessed fungicidal activities and could improve the prognosis of *A. fumigatus* keratitis by reducing fungal load and inhibiting the inflammatory response mediated by Mincle.

## Introduction

Fungal keratitis (FK) is an intractable infectious disease, and its occurrence provides a key link with agricultural corneal trauma (Khor et al., 2018; Donovan et al., 2022). The incidence of FK is significantly higher in developing countries (Brown et al., 2021). Keratohelcosis, perforation, scar formation, and endophthalmitis are common complications of FK (Durand, 2017; Sharma et al., 2022). The lack of effective antifungal drugs and poor treatment outcome in FK result in a high rate of vision loss and blindness.

*Aspergillus fumigatus* one of the most ubiquitous of the airborne saprophytic fungi, is a typical pathogen of FK. *A. fumigatus* invade and damage cornea through its virulence mechanisms, which include morphological dimorphism, adherence to host cells and tissues, hydrolytic enzymes, and toxin release (Willger et al., 2009; Sheppard, 2011; Guirao-Abad et al., 2021). In addition, *A. fumigatus* could form biofilms, which increase its pathogenicity and resistance to both host immune system and therapeutic drugs (Liu et al., 2022).

In addition to fungal damage, the excessive inflammatory response is another major cause of FK (Yang B. et al., 2021). Chitin or β-glucan on the fungal cell wall is recognized by pattern recognition receptors (PRRs) on host immune cells, mediating killing of fungal pathogens, and inducing immune cells recruitment in the infected cornea (Jaillon et al., 2013; Li et al., 2015). Immoderate inflammation can aggravate stromal damage and corneal opacity (Xu et al., 2015; Lin et al., 2017). Mincle, an important member of the PRRs family, is thought to play a promotional role in inflammatory response. It was shown that the Mincle/Syk signaling pathway could recruit neutrophils and promote downstream expression of inflammatory factors by activating mitogen-activated protein kinase (Gong et al., 2020). In addition, it stimulates inflammatory responses by maintaining M1-type macrophages (Singbartl et al., 2019). In FK, Mincle could inhibit neutrophils apoptosis and enhance expression of pro-inflammatory cytokine (Zhao et al., 2017; Yu et al., 2018). Restraint of severe immune reactions is essential for treating FK.

Isothiocyanates (ITCs) are bioactive products found in cruciferous vegetables (Soundararajan and Kim, 2018), which possess diverse biological effects, such as broad-spectrum antibacterial, antifungal, anti-inflammatory, antioxidant, and anti-tumor effects (Sønderby et al., 2010; Dias et al., 2014; Tumer et al., 2015; Alsanea and Liu, 2017). Benzyl isothiocyanate (BITC) is a member of ITCs with a benzene ring side face and short hydrogen chain. Several studies have provided evidence that the bactericidal effect of BITC is stronger than other ITCs against pathogens such as Methicillin-Resistant *Staphylococcus aureus* strains, *Candida albicans* and *Aspergillus niger*. The hydrophilic and lipophilic properties of BITC due to its chemical structure can lead to the strong antimicrobial effects as well as high adhesion efficiency and stability to bacterial components (Dias et al., 2014; Ko et al., 2016; Wang et al., 2020). BITC could regulate oxidative conditions and inflammatory processes by modulating the NF-κB pathway and the Nrf2/HO-1 axis, and inhibiting burst oxidative reactions by modulating NADPH oxidase (El Badawy et al., 2021; Sailaja et al., 2021). However, the role of BITC in FK has not been discovered yet.

In this study, we confirm that BITC possesses an antifungal effect on *A. fumigatus* and plays a protective role in *A. fumigatus* keratitis. BITC exerted antifungal effects against *A. fumigatus* by damaging cell membranes and mitochondria, inhibiting adhesion effect, and disrupting biofilm. Additionally, BITC exerts anti-inflammatory effects by downregulating Mincle expression.

## Materials and methods

### Preparation of BITC

BITC stock solution (100 mg/ml) was prepared by dissolving BITC (MEC, Shanghai, China) in dimethyl sulfoxide (DMSO, Solarbio, Beijing, China). BITC was further diluted to the concentrations of 50, 100 and 200 μg/ml for calcofluor white (CFW) staining, time-kill assay, propidium iodide (PI) uptake assay, reactive oxygen species (ROS) assay, fungal adhesion assay, and crystal violet assay. *A. fumigatus* mice model was treated with 200 μg/ml BITC, and RAW264.7 cells were treated with 3 and 6 μg/ml BITC.

### Cell culture and toxicity test

RAW264.7 cells (from Shanghai Chinese Academy of Sciences, China) were incubated in high glucose DMEM with 10% FBS at 37°C with 5% CO_2_. Human corneal epithelial cells (HCECs, from the Laboratory of the University of Xiamen, Fujian, China) were cultured in DMEM supplemented with an equal volume of Hams F12 and 5% FBS at 37°C with 5%CO_2_. RAW264.7 cells or HCECs were seeded in 96-well plates. Once cells reached 80% confluence, 100 μl of BITC (0, 3, 6, 12, 25, 50, 100, 200, 400, and 800 μg/ml) or 0.1% DMSO was added to each well and cultured for 24 h. Cell Counting Kit-8 (CCK-8; MCE) (10 μl) was added to each well, then absorbance (450 nm) was measured.

### Ocular toxicology: The Draize eye test

The experimental method is derived from earlier studies (Zhu et al., 2021). Briefly, normal mice were given 5 μl of BITC (100, 200, 400, and 800 μg/ml) into the conjunctival sac four times daily. Adverse effects of BITC on the cornea were evaluated by corneal fluorescein staining (CFS) score at 0, 1, 3, and 5 days. CFS scoring criteria refer to the Organization for Economic and Cooperative Development (OECD) grading scale for ocular irritation, which comprised corneal opacification density and area, iritis severity, conjunctival redness, edema, and secretion.

### Preparation of *Aspergillus fumigatus*

*Aspergillus fumigatus* strain (NO3.0772) was purchased from China General Microbiological Culture Collection Center. *A. fumigatus* conidia and hyphae were prepared using the previous technique (Tian et al., 2021). Conidia suspension (1 × 10^5^ CFU/ml) was used in minimum inhibitory concentration (MIC) assay, CFW staining, time-kill assay, PI uptake assay, biofilm inhibition assay, ROS assay, SEM, TEM, and fungal adhesion assay. Activated hyphae (3 × 10^8^ CFU/ml) were used to infect the cornea of mice model, and inactivated hyphae (5 × 10^6^ CFU/ml) were employed in cell experiments.

### *Aspergillus fumigatus* growth analysis

The antifungal capacity of BITC at different concentrations was tested by MIC and CFW. Conidia were seeded in a 96-well plate and subjected to BITC (0, 25, 50, 100, 200, 400, and 800 μg/ml) for 24 h. The optical density (OD) at 570 nm was measured. CFW staining was aimed to detect the antifungal of BITC to hyphae. CFW (Sigma, MO, United States) is a specific fluorescent dye for fungal cell wall chitin that binds to live fungi (Green et al., 2000). Hyphae in 6-well plates were cultured with BITC at 28°C for 6 h, the supernatant was then withdrawn and 1 ml CFW was added. A fluorescence microscope (Nikon, Tokyo, Japan, ×200) was used to capture the image.

### Time-kill assay

Fungicidal/fungistatic activities were evaluated by time-kill assay. The conidial suspension was incubated with 0.1% DMSO or BITC at 37°C, 120 rpm. The mixture (100 μl) from different times (0, 4, 6, 8, 12, and 24 h) of BITC treated conidial suspension was plated on Sabouraud agar plates and incubated at 37°C for 24 h, respectively. The colony-forming units (CFU) were counted. When compared to CFU on the mediums at 0 h, a reduction in colony count >2log10 CFU/mL was defined as fungicidal activity, and in colony count <2log10 CFU/mL was defined as fungistatic activity (Balouiri et al., 2015).

### Fungal membrane integrity assay

To determine the integrity of the fungal membrane, PI uptake assays were used after treatment with 0.1% DMSO or BITC. Experimental procedures refer to the previous method (Tian et al., 2021).

### Morphology and ultrastructure of *Aspergillus fumigatus*

Morphological and organelle changes in fungal conidia and hyphae were determined by SEM and TEM. Hyphae were germinated by *A. fumigatus* conidia at 37°C for 24 h, and then treated with 0.1% DMSO or BITC (200 μg/ml) for 8 h in 24-well plates. According to the previous method, hyphae were collected, immobilized, and dehydrated after three PBS rinses (Tian et al., 2021). Images were observed with SEM (JSM-840; JOEL Company, Japan, magnification ×2,000 and ×5,000) and TEM, respectively, (JEM-1200EX; JOEL Company, Japan, magnification ×15,000 and ×40,000).

### Measurement ROS production

ROS content in *A. fumigatus* conidia was tested by the ROS fluorometric assay kit (Elabscience, E-BC-K138-F). 10 μM 2′,7′-dichlorofluorescien diacetate (DCFH-DA) was added into conidia suspension which was treated with BITC for 6 h at 28°C before to label intracellular ROS. Conidia suspension treated by H_2_O_2_ for 2 h was regarded as the positive control. Fluorescence intensity was measured at an excitation wavelength of 500 nm and an emission wavelength of 525 nm.

### Anti-adhesion effect of BITC against *Aspergillus fumigatus* conidia

HCECs were inoculated and cultured on chambered slides (4/slide) for 24 h at 37°C. Then, BITC and *A. fumigatus* conidia were added and incubated for 4 h. PBS rinse was performed three times to get rid of non-adherent conidia. Hematoxylin–eosin (HE) staining which refer to previous articles (Jia et al., 2022) was applied to the slides. Finally, we observed and photographed specimens by microscopy (Nikon, Tokyo, Japan, ×400), and conidia adherence to HCECs was counted.

### Biofilm formation inhibition assay

Conidia were incubated in 24-well plates at 37°C for 48 h. Then, 500 μl of BITC was applied for 24 h. Each well was stained with 0.1% crystal violet (CV; Solarbio, Beijing, China) for 15 min and decolorized with 95% ethanol for 5 min. Finally, a new 96-well plate was used to transfer the supernatant and the OD value (570 nm) was measured.

### Animal models of FK

Anti-inflammatory and antifungal effects of BITC *in vivo* were evaluated using C57BL/6 mice (female, 7–8 weeks old, SPF; Pengyue Co. Ltd. Jinan, China). The modeling procedures refer to the previous method (Zhu et al., 2021). Briefly, *A. fumigatus* hyphae (5 μl; 3 × 10^8^ CFU/ml) were applied topically to the wounded corneal epithelium of anesthetized mice of left eye, a soft contact lens was placed, and eyelids were sutured. After 24 h, the eyelids sutures were removed. Then, left eyes of mice were injected subconjunctivally with 5 μl of BITC (200 μg/ml) or 5 μl of natamycin (NATA, CAS 7681-93-8; Macklin Biochemical Co. Ltd., Shanghai, China, 5 μg/ml) per day. Infected mice who administered DMSO (0.1%) in the same volume were regarded as control group. A slit-lamp microscope and clinical score (*n* = 5/group/time) were used to evaluate the severity *A. fumigatus* keratitis at 1, 3, and 5 days post infection (p.i.) (Zhu et al., 2021). Pathology of infected corneas (*n* = 6/group/time) was presented by HE staining at 3 days p.i. The work was authorized by the Research Ethics Committee of the Affiliated Hospital of Qingdao University, and the treatments given to the mice confirm to the ARVO Statement regarding the Use of Animals in Ophthalmology and Vision Research.

### Plate count

Infected corneas from 3 days p.i. were added to sterile PBS (0.1 ml) and ground into homogenate (*n* = 4/group). Sabouraud agar plates were used to incubate fungal colonies in corneal homogenates at 37°C for 24 h. The CFU on the plates was photographed and counted.

### Myeloperoxidase (MPO) assay

The amount of polymorphonuclear neutrophilic leukocytes (PMN) was quantitated by MPO kit (Njjcbio, Jiangsu, China). Corneas of FK mice (*n* = 6/group) at 3 days p.i. that treated by BITC or DMSO were tested the level of MPO in accordance with the guidelines. An enzyme-labeling instrument was used to measure absorbance (460 nm).

### RAW246.7 cell stimulation

RAW264.7 cells cultured as mentioned above were pretreated with or without BITC (3 μg/ml) for 2 h, and subsequently stimulated with inactivated *A. fumigatus* hyphae for 8 h or 24 h as required. Mincle ligand trehalose-6,6-dibehenate (20 μg/ml) (TDB Invivogen, San Diego, CA, USA) was also used to stimulate RAW264.7 cells which were pretreated with BITC for 2 h.

### Real-time polymerase chain reaction

Infected and uninfected mice corneas (*n* = 6/group/time) were added to RNAiso Plus reagent (Takara, Dalian, China) and homogenized by the TissueLyser II (28 Hz, 20 min; QIAGEN) at 3 and 5 days p.i. (Yin et al., 2022). The mRNA levels of tumor necrosis factor alpha (TNF-α), IL-1β, IL-6, and Mincle were detected in corneas supernatant. Likewise, the mRNA levels of RAW264.7 cells at 8 h after *A. fumigatus* or TDB stimulation were tests. RNAiso Plus reagent was used to extract total RNA. After spectrophotometric quantification (260 nm), the reverse transcription kit (Vazyme, Nanjing, China) synthesized the total mRNA into cDNA. RT-PCR reaction steps have been described in previous studies (Zhu et al., 2021). Table 1 displays the RT-PCR primer sequences.

**Table 1:** Nucleotide sequences of mouse primers for RT-PCR.

Gene	GenBank No.	Primer sequence (5′ - 3′)	Size (bp)
β-actin	NM_007393.3	F - GAT TAC TGC TCT GGC TCC TAG C		147
		R - GAC TCA TCG TAC TCC TGC TTG C		
IL-1β	NM_008361.3	F - CGC AGC AGC ACA TCA ACA AGA GC		111
		R - TGT CCT CAT CCT GGA AGG TCC ACG		
TNF-α	NM_013693.2	F - ACC CTC ACA CTC AGA TCA TCT T R - GGT TGT CTT TGA GAT CCA TGC		148
IL-6	NM_031168.1	F - CAC AAG TCC GGA GAG GAG AC R - CAG AAT TGC CAT TGC ACA AC		141
Mincle	XM_017321688	F - ACT GAC AGA CCA GGT GGT GGA G		198
		R - TCA CAA ATC CAA GGC ATA CTG TAG A		

### Western blot

Mice corneas (*n* = 5/group/time) for 3 and 5 days p.i. and RAW264.7 cells after stimulation of inactivated hyphae or TDB for 24 h were collected for detection of Mincle protein levels, which were fully lysed in RIPA solution (Solarbio, Beijing, China) with 1% PMSF and Phosphatase inhibitor. Concentrations of protein were then determined by a BCA Assay Kit (Elabscience). Separated proteins in PVDF (polyvinylidene fluoride) membranes (Solarbio) were transferred from SDS-PAGE glue. After 2 h’s blocking with a blocking buffer (Beyotime Biotechnology, Shanghai, China), the PVDF membranes containing proteins were incubated with goat anti-mouse Mincle (1:500, Santa Cruz, CA, United States) antibody or goat anti-rabbit β-actin (1:2000, Elabscience) antibody overnight at 4°C and with the corresponding secondary antibody (1:2000, Elabscience) for 2 h at 28°C. Chemiluminescence (ECL; Thermo Fisher Scientific, United States) was used to inspect the blots.

### Enzyme-linked immunosorbent assay

Corneas (*n* = 5/group/time) were collected, homogenized, and centrifuged (12,000 rpm, 5 min) according to the reported literature at 3 and 5 days p.i. (Yin et al., 2022). RAW264.7 cell cultures supernatants (*n* = 6/group) were harvested at 24 h after stimulation. Protein expression of TNF-α and IL-1β was measured by ELISA kits (R&D system, United States). Experimental procedures were performed according to the manufacturer’s instructions. The absorbance (450 nm and 570 nm) was measured.

### Immunofluorescence staining

RAW264.7 cells were seeded and treated by BITC for 24 h in chambered slides (4/slide) plates. After fixation with paraformaldehyde, chambered slides (4/slide) containing cells were incubated overnight with Mincle (1:200; Santa Cruz, CA, United States) primary antibody and FITC-conjugated IgG goat anti-rat secondary antibody (1:200; Elabscience) for 1 h, and DAPI (ready-to-use, Solarbio) to label the cell nucleus. Finally, fluorescent microscopy (Zeiss Axio Vert; magnification ×400) was used to observe and take pictures, and the OD in the pictures was analyzed by Image J software.

### Statistical analysis

Data were presented as mean ± standard deviation (SD) and analyzed using GraphPad Prism 8 software. The Mann–Whitney U test examined differences in clinical scores. Unpaired two-tailed Student’s *t*-test was used to analyze the data from MPO assay. Data from CCK-8 assay, MIC assay, time-kill assay, biofilm assay, RT-PCR, Western blot, ELISA, plate count, and IFS were analyzed by one-way analysis of variance (ANOVA) test. Further two-by-two comparison was performed using Bonferroni analysis. *p* < 0.05 was considered difference significant. Three times each experiment was repeated.

## Results

### Safety evaluation of BITC *in vivo* and *in vitro*

**Figure 1:**
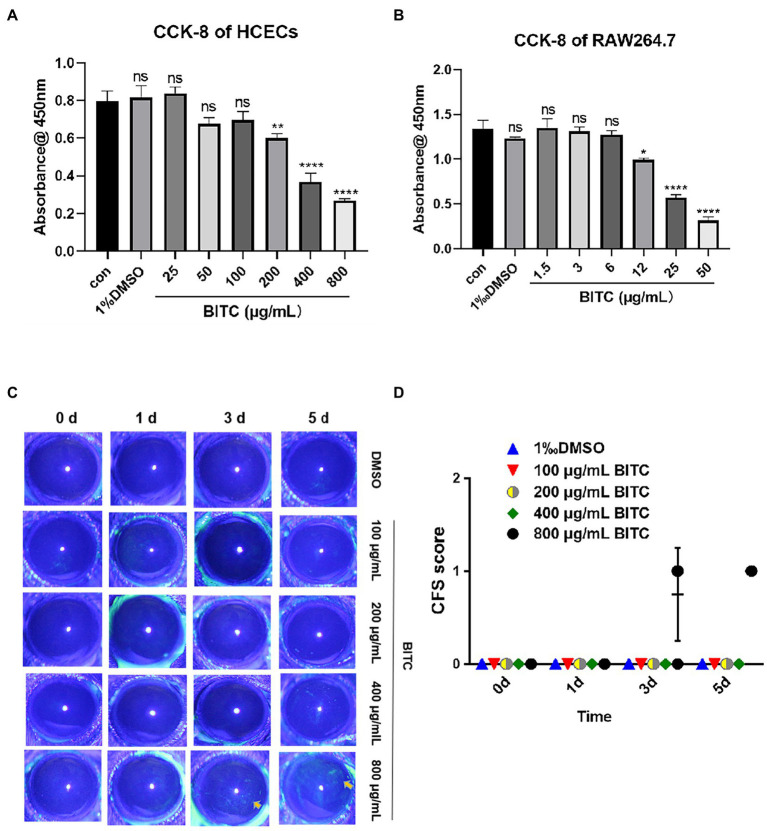
Safety of different concentrations of BITC on host cells and cornea. Cell viability of HCECs **(A)** and RAW264.7 **(B)** after treatment by BITC at different concentrations for 24 h. Toxicity to mice cornea were evaluate by CFS **(C)** and CFS scores **(D)**. Values represent as means ± SD (*p < 0.05, **p < 0.01, ***p < 0.001, ****p < 0.0001).

To explore the toxicity of BITC and the optimal concentrations for subsequent research on HCECs and RAW264.7 cells, we performed CCK-8 experiments with a range of concentrations of BITC (0, 3, 6, 12, 25, 50, 100, 200, 400, and 800 μg/ml). Compared with control groups, the absorbance remained the same in HCECs (Figure 1A) after treated with 100 μg/ml BITC, and in RAW264.7 cells (Figure 1B) after treated with 6 μg/ml BITC for 24 h, indicating the above-mentioned concentration of BITC is safe for HCECs and RAW264.7 cells. Next, we evaluated the performance of BITC on mice corneas by the Draize eye test. No anterior segment damage (conjunctiva, cornea, anterior chamber, and iris) and corneal staining tested by fluorescein sodium were discovered after BITC treatment (100–400 μg/ml) for 1, 3, and 5 days, as well as after 800 μg/ml BITC treatment for 1 day (Figures 1C,D). Despite the fact that conjunctival congestion and corneal neovascularization were not found, CFS displayed a small amount of punctate staining of the cornea after receiving BITC treatment at 800 μg/ml for 3 and 5 days.

### Killing effect of BITC on *Aspergillus fumigatus*

**Figure 2:**
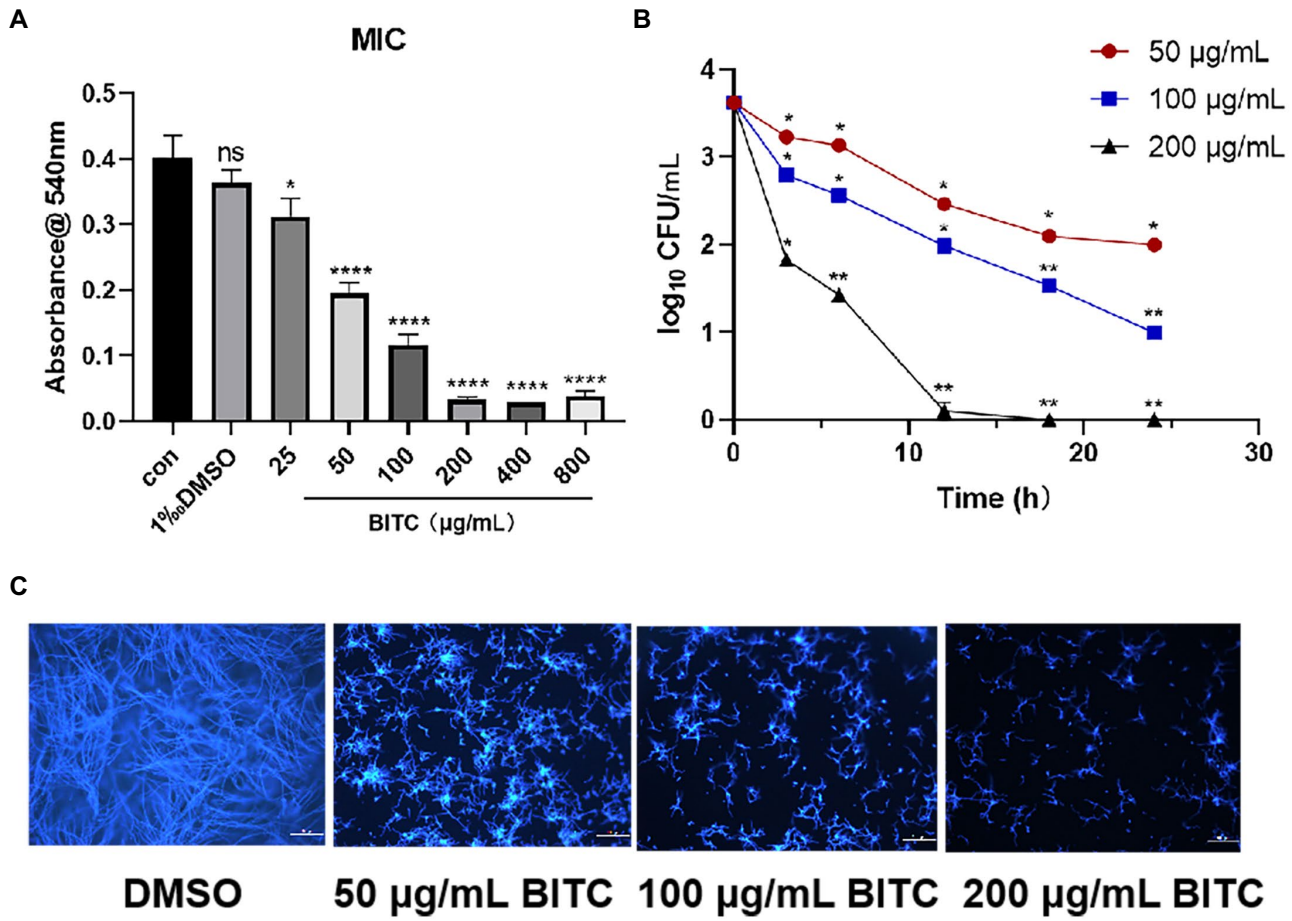
Fungicidal effect of BITC against *A. fumigatus*. The effect of BITC on growth and germination of *A. fumigatus* for 24 h **(A)**. Time-kill curves **(B)** for *A. fumigatus* exposed to BITC (50, 100 and 200 μg/ml) at different time point (* < 2log10 CFU/mL, ** > 2log10 CFU/mL). Calcofluor white staining **(C)** showed surviving *A. fumigatus* hyphae under treatment with 0.1% DMSO and BITC (50, 100 and 200 μg/ml), magnification ×200. Values represent as means ± SD (*p < 0.05, **p < 0.01, ***p < 0.001, ****p < 0.0001).

The antifungal effect of BITC at different concentrations was evaluated during the growth of *A. fumigatus*. MIC results showed that the growth and germination of *A. fumigatus* were both inhibited by treatment of BITC (25 μg/ml) for 24 h (*p* < 0.05). As concentration of BITC raised, the inhibition rate increased until it reached 100% at 200 μg/ml of BITC (Figure 2A). Data from the time-kill assay demonstrated that the amount of *A. fumigatus* CFU was significantly decreased in BITC-treated groups at 50, 100, or 200 μg/ml compared to the control group at each time point (Figure 2B). As treatment time extended, the number of CFU at 50 μg/ml of BITC (red line) and 100 μg/ml of BITC (blue line) declined steadily in a concentration-dependent manner. While the number of the colonies was decreased rapidly by BITC (200 μg/ml; black line) at 3 h, and almost 0 at 18 h. Images of CFW showed intuitively the surviving hyphae (blue fluorescence; Figure 2C) after treatment of BITC at 50, 100, and 200 μg/ml. The hyphae treated with DMSO were more robust and dense, while BITC treatment significantly reduced the amount of surviving hyphae that were slender and short. The number of viable hyphae decreased with increasing BITC concentration.

### Antifungal mechanism of BITC on *Aspergillus fumigatus*

**Figure 3:**
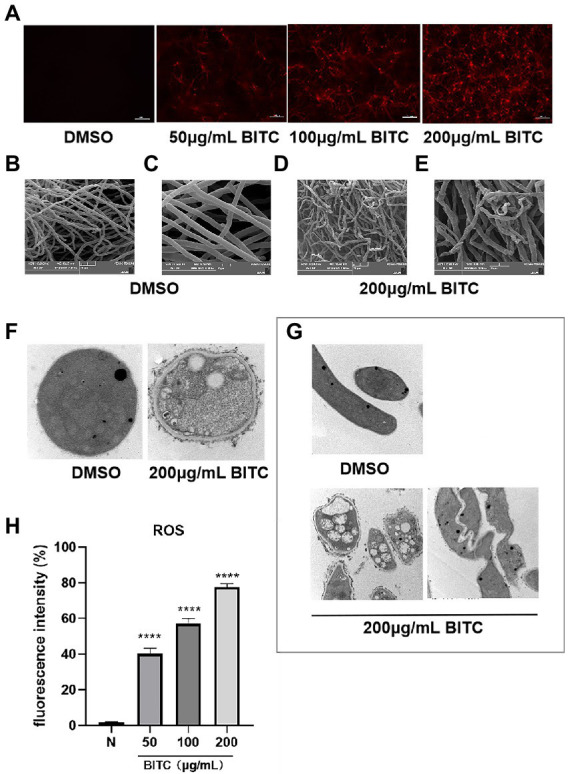
Killing mechanism of BITC on *A. fumigatus.* PI staining **(A)** for membrane integrity of *A. fumigatus* exposed to 0.1% DMSO and BITC (50, 100 and 200 μg/ml), magnification ×200. SEM images of *A. fumigatus* hyphae morphology after treatment with 0.1% DMSO, magnification ×2,000 **(B)** and ×5,000 **(C)** and 200 μg/ml BITC, magnification ×2,000 **(D)** and ×5,000 **(E)**. TEM images of *A. fumigatus* conidia, magnification ×40,000 **(F)** and hyphae, magnification ×15,000 **(G)** after treatment with 0.1% DMSO and 200 μg/ml BITC. Effects of BITC (50, 100, and 200 μg/ml) on the increase of ROS **(H)** in *A. fumigatus* conidia. Values represent as means ± SD (****p < 0.0001).

PI staining showed negative fluorescence in *A. fumigatus* treated with 0.1% DMSO, whereas in *A. fumigatus* treated with BITC at concentrations of 50, 100, and 200 μg/ml, red fluorescence was observed (Figure 3A). Additionally, the fluorescence of PI was increased by BITC in a dose-dependent manner. In SEM images, there were no apparent morphological alterations in untreated hyphae, which were long and with smooth surface (Figures 3B,C). Contrarily, hyphae exposed to BITC (200 μg/ml) for 8 h exhibited shrinkage and deformation, and were broken into fragments (Figures 3D,E). TEM images (Figures 3F,G) revealed the cellular ultrastructure in DMSO-treated and BITC-treated conidia and hyphae of *A. fumigatus.* A normal cellular ultrastructure with homogenous cell wall thickness, cytoplasmic density, and normal mitochondrial morphology was observed in untreated conidia and hyphae of *A. fumigatus.* However, it could be seen detachment of cell membrane from the cell wall and loss of the integrity of cell membrane in both conidia and hyphae after BITC (200 μg/ml) treatment for 8 h. Additionally, conidia (Figure 3F) showed cell wall thickening, loose cytoplasmic and disrupted mitochondrial morphology, and hyphae (Figure 3G) displayed distorted and shriveled morphology and swollen mitochondria with destroyed cristae structures. The level of ROS in *A. fumigatus* conidia was measured by fluorescent probe DCFH-DA. As shown in Figure 3H, treatment of BITC (50, 100, and 200 μg/ml) for 6 h caused a sustained increase in the level of ROS. With the increase of BITC concentration, the accumulation of ROS in conidia was upregulated significantly (*p* < 0.05).

### Inhibition of conidia adhesion and biofilm by BITC

**Figure 4:**
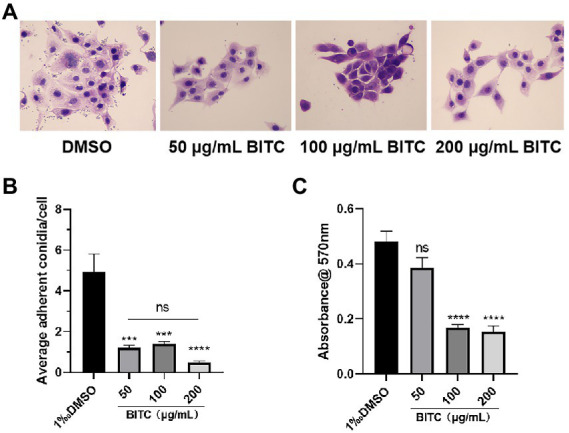
BITC inhibited *A. fumigatus* conidia adhesion and biofilm. HE staining images **(A)** of adherent *A. fumigatus* conidia to HCECs after 0.1% DMSO and BITC (50, 100 and 200 μg/ml) treatment (magnification ×400), and the quantitative diagram is shown **(B)**. The inhibition of biofilms **(C)** under the treatment of BITC (50, 100 and 200 μg/ml) and 0.1% DMSO. Values represent as means ± SD (***p < 0.001, ****p < 0.0001).

*Aspergillus fumigatus* conidia in the control group had strong adherence to HCECs, with about 5 conidia adhering to each HECE. Only a small number of conidia stuck to HCECs under treatment of BITC (50, 100, 200 μg/ml) after 4 h (Figures 4A,B). Seldom conidia adherence could be observed under the treatment of BITC (200 μg/ml). The inhibition of BITC on mature biofilms was quantified by measuring the absorbance of CV staining. Absorbances of stained biofilms were decreased significantly by BITC at 100 and 200 μg/ml (Figure 4C; *p* < 0.001).

### BITC is protective on *Aspergillus fumigatus* keratitis in mice

**Figure 5:**
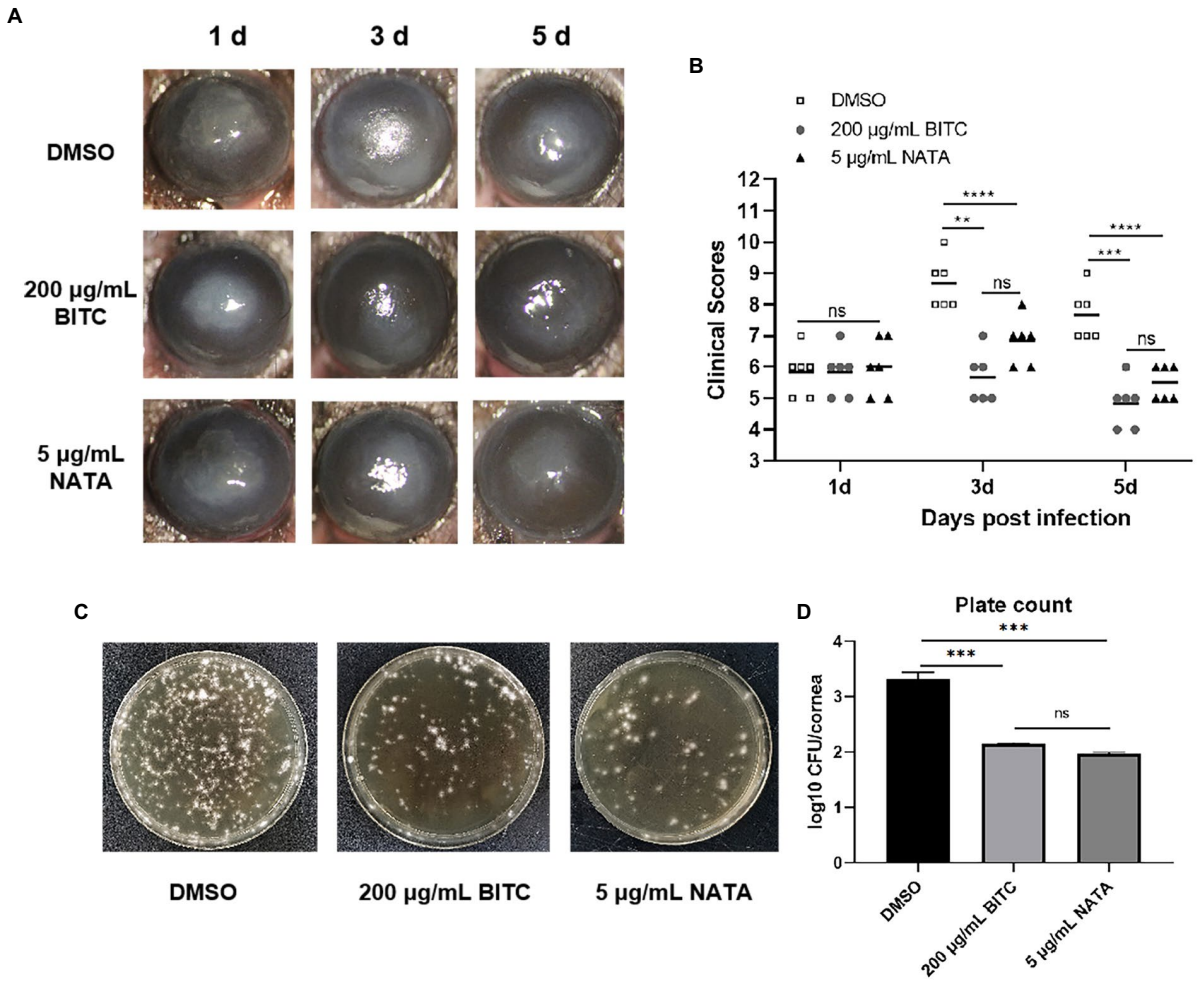
BITC is protective on *A. fumigatus* keratitis in mice. Slit-lamp photography **(A)** and clinical scores **(B)** for disease severity of *A. fumigatus* keratitis. The fungal load in corneas **(C)** and quantitative diagram **(D)** in *A. fumigatus* keratitis at 3 days p.i. Values represent as means ± SD (*p < 0.05, **p < 0.01, ***p < 0.001, ****p < 0.0001).

BITC, NATA, or DMSO were applied topically in order to evaluate the protective effect in *A. fumigatus* keratitis mice of BITC. No differences were observed in corneal ulceration and opacity demonstrated by slit-lamp photography (Figure 5A) at 1 day p.i. While the 200 μg/ml BITC (*p* < 0.01, *p* < 0.001) and 5 μg/ml NATA groups (*p* < 0.0001, *p* < 0.0001) alleviated corneal ulceration and opacity (Figure 5A), and obtained lower clinical scores (Figure 5B) compared to the 0.1%DMSO group at 3 and 5 days p.i. In addition, plate counting (Figures 5C,D) experiments showed that both 200 μg/ml BITC (*p* < 0.001) and 5 μg/ml NATA (*p* < 0.001) considerably decreased fungal load in the infected corneas when compared to the DMSO-treated group and no difference between the BITC and NATA groups (*p* > 0.05).

### BITC inhibits inflammatory response

**Figure 6:**
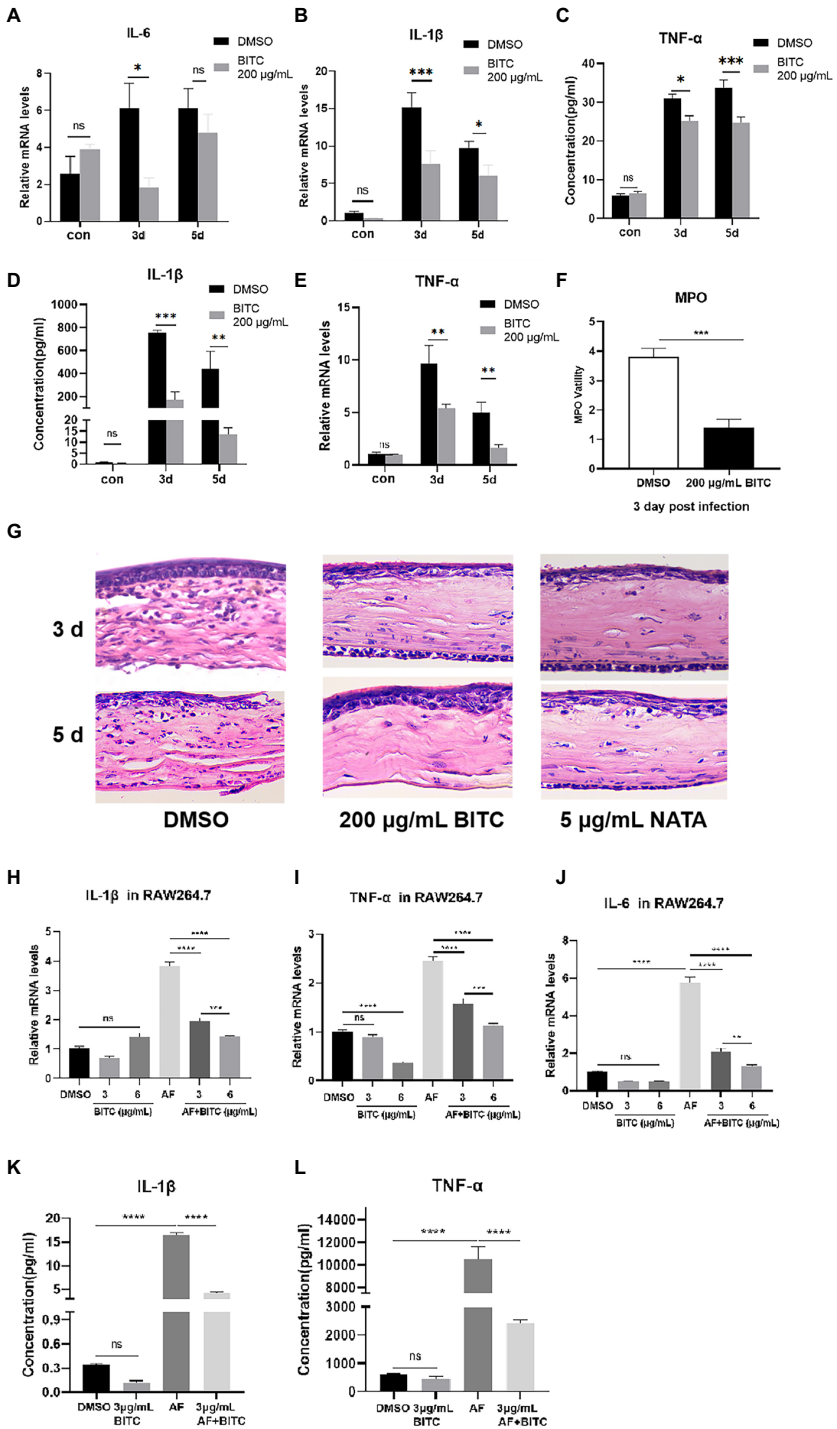
BITC inhibits inflammatory response *in vivo* and *in vitro*. RT-PCR results for IL-6 **(A)**, IL-1β **(B)**, TNF-α **(C)** and ELISA results for IL-1β **(D)**, TNF-α **(E)** at 3, 5 days p.i. in normal and *A. fumigatus*-infected mice cornea under BITC (200 μg/ml) or 0.1% DMSO treatment. Levels of MPO **(F)** in *A. fumigatus* cornea under BITC (200 μg/ml) or 0.1% DMSO treatment. HE staining **(G)** of infected corneas under 0.1% DMSO, BITC (200 μg/ml) or NATA (5 μg/ml) treatment at 3 days p.i. (magnification ×400). The mRNA levels of IL-1β **(H)**, TNF-α **(I)**, and IL-6 **(J)** at 8 h and protein levels of IL-1β **(K)** and TNF-α **(L)** at 24 h in RAW264.7 cells infected by *A. fumigatus*. Values represent as means ± SD (*p < 0.05, **p < 0.01, ***p < 0.001, ****p < 0.0001).

Next, we investigated how BITC treatment affected corneal immune cell infiltration and expression of pro-inflammatory cytokines after infection. The expression of pro-inflammatory cytokines in mice corneas was tested by RT-PCR and ELISA. PCR data showed decreased IL-6 mRNA in the 200 μg/ml BITC-treated group than in DMSO-treated group at 3 days p.i. (Figure 6A; *p* < 0.05). Moreover, our results revealed that the mRNA expression of IL-1β (Figure 6B; *p* < 0.001, *p* < 0.5) and TNF-α (Figure 6C; *p* < 0.01, *p* < 0.01) was considerably decreased following BITC treatment compared with DMSO-treated infected corneas at 3 and 5 days p.i. Consistently, the protein expression of IL-1β (Figure 6D; *p* < 0.001, *p* < 0.01) and TNF-α (Figure 6E; *p* < 0.05, *p* < 0.001) in the 200 μg/ml BITC-treated group was lower than DMSO-treated group. MPO levels were lower in 200 μg/ml BITC-treated mice corneal than DMSO-treated group at 3 days p.i. (Figure 6F; *p* < 0.001). In addition, HE staining showed a large infiltration of immune cells in the corneal stroma of FK at 3 and 5 days p.i. (Figure 6G). While corneal infiltration of immune cells in the 200 μg/ml BITC and 5 μg/ml NATA treatment groups were alleviated at 3 and 5 days p.i. (Figure 6G). In infected corneas, pro-inflammatory cytokines were downregulated by BITC, and the effect of BITC in RAW264.7 cells stimulated by *A. fumigatus* was verified further. RT-PCR results manifested that the expression of IL-1β (Figure 6H; *p* < 0.0001, *p* < 0.0001), TNF-α (Figure 6I; *p* < 0.001, *p* < 0.001) and IL-6 (Figure 6J; *p* < 0.0001, *p* < 0.0001) mRNA was significantly reduced by BITC (3 and 6 μg/ml) after 8 h of *A. fumigatus* stimulation in a concentration-dependent manner. Protein levels of IL-1β (Figure 6K; *p* < 0.0001) and TNF-α (Figure 6L; *p* < 0.0001) were similarly noticeably reduced in the BITC-treated (3 μg/ml) group after 24 h.

### BITC downregulates Mincle to inhibit inflammatory response

**Figure 7:**
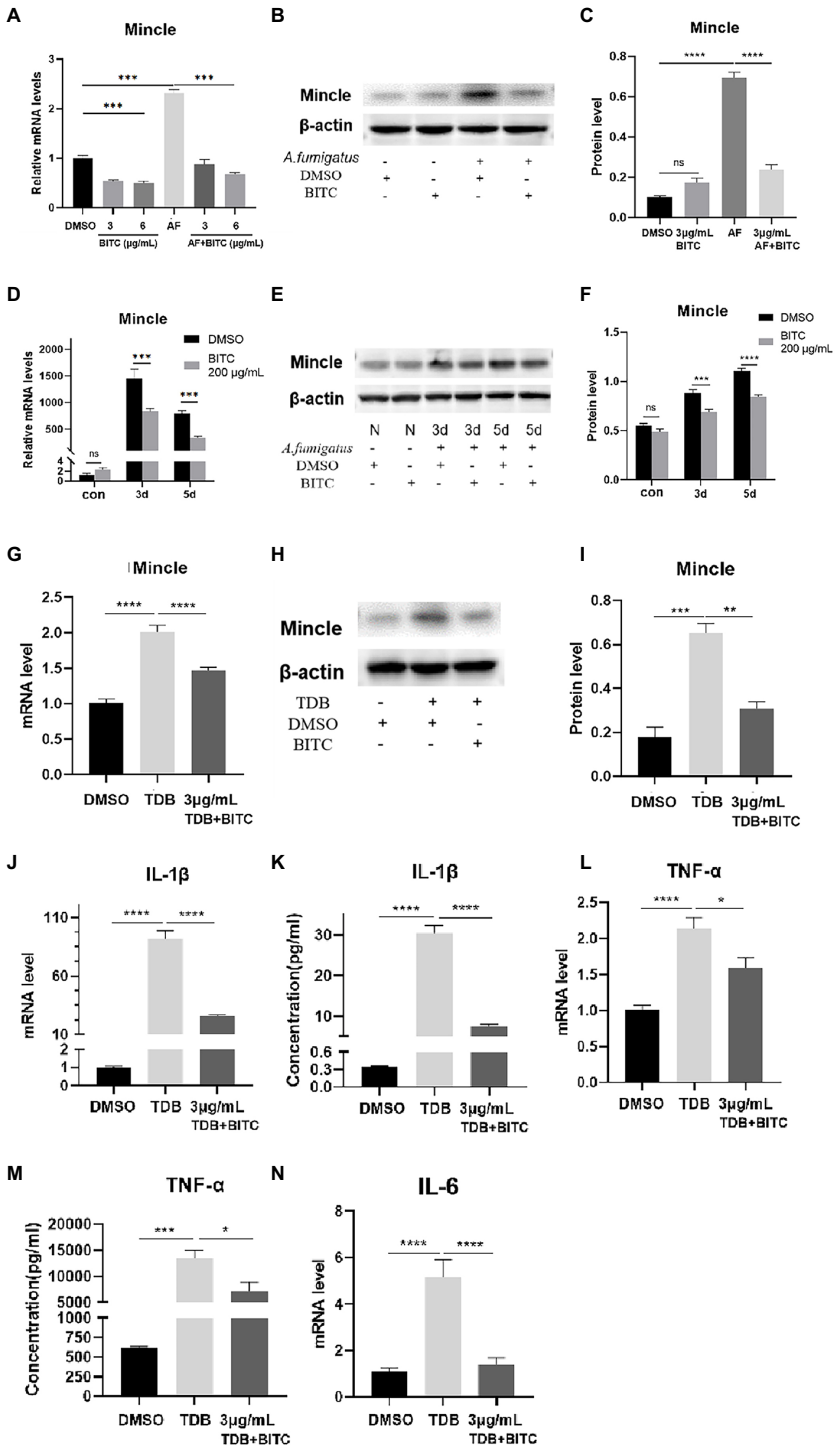
BITC downregulates Mincle to inhibit inflammatory response. The mRNA expression of Mincle (**A**) decreased after treatment of BITC (3 and 6 μg/ml) in *A. fumigatus* stimulated RAW264.7 cells. Mincle protein level was also significantly repressed by BITC (3 μg/ml) treatment (**B**,**C**) in RAW264.7 cells. Mincle mRNA expression (**D**) and protein expression (**E**,**F**) were decreased in BITC-treated (200 μg/ml) corneas infected by *A. fumigatus* at 3 and 5 days p.i. The expression of Mincle mRNA (**G**) and protien (**H**,**I**) was decreased after treatment of BITC (3 μg/ml) in TDB-stimulated RAW264.7 cells. Effects of BITC on TDB-stimulated RAW264.7 cells RT-PCR and ELISA results for IL-1β (**J**,**K**), TNF-α (**L**,**M**), and IL-6 (**N**) in TDB-stimulated RAW264.7 cells treated with BITC (3 μg/ml) or 0.1% DMSO. Values represent as means ± SD (*p < 0.05, **p < 0.01, ***p < 0.001, ****p < 0.0001).

**Figure 8:**
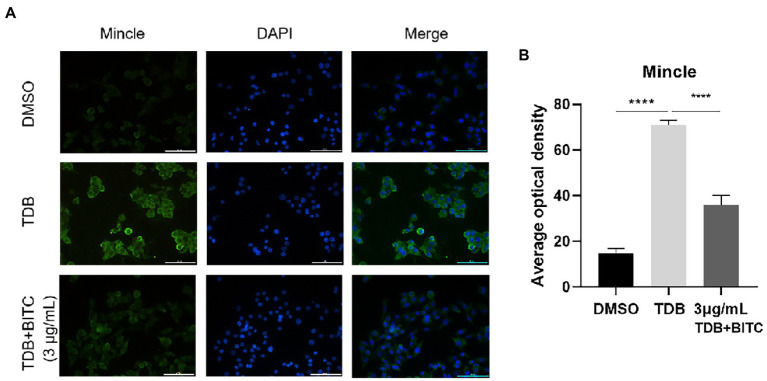
The immunofluorescence staining **(A)** labeled Mincle protein in RAW264.7 cells stimulated with TDB and treated by DMSO or BITC. Quantitative analysis of Mincle protein levels **(B)** was conducted by Image J. Values represent as means ± SD. (****p < 0.0001).

The data from RT-PCR and western blot indicated that the mRNA and protein levels of Mincle were elevated notably after *A. fumigatus* infection in RAW264.7 cells (Figures 7A–C; *p* < 0.001, *p* < 0.0001), and Mincle expression reduced under the treatment of BITC (3 μg/ml and 6 μg/ml) (*p* < 0.001, *p* < 0.0001). The same result was observed in infected corneas that BITC (200 μg/ml) significantly downregulated the Mincle mRNA (Figure 7D; *p* < 0.001, *p* < 0.001) and protein (Figures 7E,F; *p* < 0.001, *p* < 0.0001) levels compared to the control group at 3 and 5 days p.i. RAW264.7 cells were stimulated by TDB, a Mincle specific agonist, to further define the role of BITC on Mincle. Our results revealed that treatment of BITC (3 μg/ml) suppressed the elevated expression of Mincle (Figures 7G–I; *p* < 0.0001, *p* < 0.001), IL-1β (Figures 7J,K; *p* < 0.0001, *p* < 0.0001), TNF-α (Figures 7L,M; *p* < 0.05, *p* < 0.05), and IL-6 (Figure 7N; *p* < 0.0001) at mRNA and protein levels that stimulated by TDB. IFS was used to label Mincle protein in RAW264.7 cell stimulated by TDB for 24 h. The results further confirmed that 3 μg/ml BITC effectively decreased the level of Mincle protein that stimulated by TDB (Figures 8A,B; *p* < 0.001).

## Discussion

FK is a complex, refractory keratopathy that may be attributed to the limited efficacy of current antifungal drugs and excessive inflammatory response (Ung et al., 2019; Prajna et al., 2022). BITC is a secondary metabolite extracted from the cruciferous species and has received attention for its broad pharmacological properties including antimicrobial, anti-inflammatory, and antioxidant activities. The antifungal effects and mechanisms of BITC and other ITCs including allyl ITC (AITC), phenylethyl ITC (PEITC) and sulforaphane (SFN) on different fungi, such as *A. flavus*, *A. niger*, *A. carbonarius*, *C. albicans*, *Trichophyton mentagrophytes*, etc., have been studied, and their antifungal ability has been compared (Pereira et al., 2020; Yang R. B. et al., 2021; Hareyama et al., 2022). Studies of ITCs in inflammation have revealed that ITCs are potent activators of the Nrf2, which is an important regulator of oxidative stress and inflammation (Ernst et al., 2011; Wagner et al., 2013). Moreover, BITC and SFN suppress LPS-induced inflammatory responses by inhibiting NF-κB (Heiss et al., 2001; Lee et al., 2009). BITC possessed anti-inflammatory activity in lipopolysaccharide-stimulated BV2 microglial cells and in several acute inflammation models (Lee et al., 2016; Ibrahim et al., 2018). In this study, we identified that BITC exerts antifungal and anti-inflammatory activities during *A. fumigatus* keratitis.

Our study demonstrated that BITC in the safe concentration range was suppressed the germination and growth of *A. fumigatus* in a time-and concentration-dependent manner. Fungicidal mechanisms of BITC were further explored. The results of both CWF and SEM demonstrated the destructive effect of BITC on the hyphae of *A. fumigatus*. Hypha is one of the major virulence of *A. fumigatus* which facilitates attachment to the host cell and impairs the activation of immune cells (Riquelme et al., 2018; McBride et al., 2019). Many laboratories have also investigated how BITC affects the hyphal morphology of fungi such as *C. albicans* and *A. ochraceus*, and results from SEM and Raman imaging spectroscopy have shown that BITC killed and structurally damaged hyphae (Clemente et al., 2016; Pereira et al., 2020). It has been demonstrated that the membrane integrity of *A. alternata* was virtually entirely compromised by BITC (Wang et al., 2020). Our findings are in line with the study mentioned above. In *A. fumigatus* conidia treated with BITC, TEM images revealed a loss of cell membrane integrity. Results from PI uptake experiments supported the aforementioned finding. Studies revealed that BITC treatment reduced the lipid components in the conidia and hyphae of *A. ochraceus* and that BITC could covalently bind to aminophospholipids and phosphatidylethanolamine in the cell membrane (Clemente et al., 2016; Nakamura et al., 2019). In addition, ITCs can react with nucleophiles such as thiol groups in proteins (Krell et al., 2021). The mechanism of BITC damage to cell membranes may be involved in a reaction with lipid components and proteins on the cell membrane. We then explored the effect of BITC on fungal ROS production and mitochondrial disruption. Redox homeostasis is essential for mitochondria, cells, and organisms to function properly (Zorov et al., 2014). Previous studies have shown that BITC can disrupt redox reactions of cells through various mechanisms, such as inhibition of glutathione reductase and cytochrome c oxidase, as well as disruption of mitochondrial function, leading to cell death or apoptosis initiation (Li et al., 2020; Henklewska et al., 2021). In our study, BITC induced ROS within fungi conidia in a concentration-dependent manner. TEM images also revealed the disorganization of organelles and disruption and disintegration of mitochondrial morphology after BITC treatment. Excessive ROS would attack and disrupt the mitochondrial membrane, which can lead to ROS and free radicals outbreaks and cellular injury (Zorov et al., 2014). Consistently, it has been reported that BITC induced significant accumulation of ROS and caused mitochondrial membrane potential collapse on *C. albicans* (Dufour et al., 2013; Zhang and Chen, 2017). Thus, we speculated that induction of rapid ROS overproduction and damage of mitochondria were associated with the fungicidal mechanisms of BITC. Biofilm formation is regarded as a major virulence factor related to fungal resistance and pathogen transmission (Gilbert et al., 2002). The results of CV staining indicated that BITC is capable to rupture the mature biofilms of *A. fumigatus*. Additionally, a study found that BITC significantly reduced *Salmonella typhimurium* biofilm development (Niu et al., 2020). This is coherent with our findings. In addition, we demonstrated that BITC inhibits the ability of *A. fumigatus* to adhere to HCECs. These findings allow us to conclude that BITC exerts a fungicidal effect on *A. fumigatus* in multiple ways, including damage to cell membranes, mitochondria, biofilms, and adhesion activities.

BITC was employed topically in corneas of *A. fumigatus*-infected mice to verify the therapeutic effect. Treatment with BITC had a positive impact on disease outcome, as indicated by clinical score and slit-lamp photography, and reduced corneal fungal load. These findings implied that BITC was effective in limiting the progression of FK. In addition, the efficacy of BITC on *A. fumigatus* keratitis was observed to be comparable to NATA under the conditions of this experiment.

As already mentioned in the introduction, exacerbation of corneal opacity and edema is significantly influenced by an unchecked inflammatory response. In the cisplatin-induced acute renal injury murine model, BITC treatment downregulated the expression of TNF-α and IL-1β and has been verified to possess potent nephroprotective and anti-inflammatory effects (Ibrahim et al., 2018). Therefore, we speculate that in addition to its antifungal effect, BITC may have another protective effect on FK by inhibiting the inflammatory response. To confirm this theory, the expressions of pro-inflammatory cytokines were evaluated in RAW264.7 cells and corneas of *A. fumigatus* keratitis. Data provided evidence that BITC administration greatly reduced the production of TNF-α, IL-1β and IL-6 stimulated by *A. fumigatus* both *in vivo* and *in vitro*. In addition, infiltration of inflammatory cells in BITC-treated infected corneas was significantly reduced. These findings are in line with a study showing that the contents of pro-inflammatory cytokines and infiltration of inflammatory cells in the submucosa were reduced by BITC in the acute renal injury mice model (El Badawy et al., 2021). Therefore, our results indicate that BITC limits inflammation by inhibiting inflammatory cell infiltration and reducing downstream inflammatory cytokines.

However, how BITC suppresses the inflammatory response in FK remains unknown. The intrinsic immune response is triggered and amplified by PRRs, according to earlier research (Leal et al., 2010). Studies have revealed that Mincle plays a significant role in FK by recruiting neutrophils and promoting expression of pro-inflammatory cytokines (Yu et al., 2018). In current investigation, Mincle was elevated notably after in RAW264.7 cells and corneas infected by *A. fumigatus*, which were suppressed by BITC. Next, we explored whether BITC could inhibit the inflammatory response in RAW264.7 cells induced by TDB. Data illustrated a significant increase in levels of TNF-α, IL-1β and IL-6 when Mincle was upregulated by TDB. Meanwhile, expressions of Mincle and pro-inflammatory factors were suppressed after treatment of BITC. These findings suggest that BITC improves the prognosis of FK and exhibits its anti-inflammatory abilities by specifically inhibiting Mincle expression.

In conclusion, this study demonstrated that BITC improves the prognosis of FK through both antifungal and anti-inflammatory approaches. BITC exerts fungicidal effects to *A. fumigatus* by disrupting cell membranes and mitochondria, boosting the production of ROS, obstructing fungal adhesion, and rupturing biofilms. In addition, BITC lessens the infiltration of inflammatory cells and the expression of inflammatory factors by suppressing the expression of Mincle. Our study suggests that BITC displays potential therapeutic function in the treatment of FK.

## Data availability statement

The datasets presented in this study can be found in online repositories. The names of the repository/repositories and accession number(s) can be found in the article/supplementary material.

## Ethics statement

The animal study was reviewed and approved by The Affiliated Hospital of Qingdao University.

## Author contributions

WY designed and performed the experiments, analyzed the data, and wrote the paper. LG analyzed the data, and wrote the paper. CL designed the experiments, analyzed the data and wrote the paper. YW performed the experiments. JL analyzed the data. LZ analyzed the data. QW analyzed the data. YQ performed the experiments. WD performed the experiments. MY performed the experiments. GZ designed the experiments. All authors contributed to the article and approved the submitted version.

## Funding

This work was financially supported by the National Natural Science Foundation of China (Nos. 82171029, 81870632 and 81800800), China Postdoctoral Science Foundation (Nos. 2020M672000), and the Taishan Scholars Program (Nos. ts201511108, tsqn202103188 and tsqn201812151).

## Conflict of interest

The authors declare that the research was conducted in the absence of any commercial or financial relationships that could be construed as a potential conflict of interest.

## Publisher’s note

All claims expressed in this article are solely those of the authors and do not necessarily represent those of their affiliated organizations, or those of the publisher, the editors and the reviewers. Any product that may be evaluated in this article, or claim that may be made by its manufacturer, is not guaranteed or endorsed by the publisher.
